# Molecular docking analysis of modified gedunin from neem with snake venom enzymes

**DOI:** 10.6026/97320630017776

**Published:** 2021-09-30

**Authors:** Priya Dagar, Abha Mishra

**Affiliations:** 1 Department of Biochemical Engineering, IIT (BHU), UP State, Varanasi - 221005, India

**Keywords:** Gedunin, Inhibitors, Snake venom enzymes, plant metabolite

## Abstract

Snakebites are a problem due to the increasing number of deaths and permanent disabilities. There is currently a shortage of antidotes for snakebite. The existing antibody antidote, produced from horse/sheep plasma/sera is expensive, species-dependent, and causes fatal side effects. Therefore, it is of interest use of natural flavonoid named gedunin from the Azadirachta indica (Neem) plant species to combat snakebites. Thus, we show the molecular docking analysis of gedunin (C26H31N2O6F) with enzymes (common in snake species) such as 5-nucleotidase, acetyl cholinesterase, L-aao, metalloproteinase, serine, thrombin and phospholipase A2. The modified gedunin in the enzyme pocket showed improved pharmacological properties for further consideration in combating snakebites.

## Background:

Snakebite has become a generally neglected public health issue, responsible for more than 137,880 deaths and poisoning around two million people annually worldwide. Women, children, the poor, and farmers are at a higher risk with weak and sparse medical resources such as anti-serum [5]. The side effects of antiserum are serum sickness or delayed hypersensitivity and local tissue damage due to non-immunoglobulin proteins in the antiserum [3]. However, plant extracts have been effective against snake venom since ancient times and retain growing interest due to abundance and safety [11]. A study suggests that Azadirachta indica have compounds such as steroids, alkaloids, triterpenoid, tetraterpenoid, tannins, phenols, pterocarpans, and glycosides effective against snake venom by neutralizing multiple toxins and enzymes (hydrolases, proteases, phospholipase, ATPase, transaminase, Nucleotidase) poison [13]. Gedunin is a tetra nortriterpenoid isolated from the neem tree (Azadirachta indica, Meliaceae) used in traditional medicine to treat malaria and other infectious diseases. Moreover, Gedunin from Neem has shown anti-proliferative activity against various cancer cell lines, including prostrate, colon, and ovarian cancers. Gedunin is a robust and thiol-reactive electrophile that activates the heat shock response [13]. Therefore, it is of interest to show the molecular docking analysis of a natural flavonoid named gedunin from the Azadirachta indica (Neem) plant species with enzymes (common in snake species) such as 5-nucleotidase, acetyl cholinesterase, L-aao, metalloproteinase, serine, thrombin and phospholipase A2. The modified gedunin in the enzyme pocket showed improved pharmacological properties for further consideration in combating snakebites.

## Materials & Methods:

### The ICM method:

The ICM software was used to perform flexible ligand docking with map grid calculated for the enzyme active site pocket. The Monte Carlo method used in the ICM follows a procedure [5] where random movement for the conformational variable of the ligand in the enzyme pocket is possible [3]. Calculation of the desolvation energy followed by selecting the minimized conformation using the Metropolis method was completed. The user-defined multiplier was kept at three according to the number of ligand rotational bonds. The calculated grid maps for hydrogen bonds, van der Waals bonds, electrostatic and hydrophobic potentials reduced the time required for ligand sampling. This generated 0.5 Å grid spacing maps at the ligand-binding site. The global optimization of the energy carried out requires a high dimensionality is reduced in the ICM by assigning internal coordinates to each atom [14].

### Ligand preparation:

The ligand was developed using the ICM object by removing water followed by optimization of hydrogen, his-pro-asn-gln-cys. This conformational analysis was completed outside the pocket of the enzyme (receptor). The modified form of Gedunin (C26H31N2O6F) is the ligand for the study (Figure 1 and Figure 2 - see PDF).

### Receptor preparation:

The PDB structure coordinates for 5-nucleotidase, acetyl cholinesterase; L-aao, metalloproteinase, serine, thrombin and phospholipase A2 were downloaded from RCSB PDB and processed using the ICM-Pro Molsoft-Software [9][12].

### Molecular docking:

Molecular docking analysis of snake venom enzymes with modified gedunin from neem was completed using ICM-Pro Molsoft-Software (Tables 1, 2, 3 - see PDF). The ICM score is the sum of the ligand-target Vander-Waals interactions and the internal force field energy of the ligand (DE IntFF 5), hydrogen bond interactions (DE HBond Tor), free energy changes due to conformational energy loss with ligand binding (TDS), solvation of electrostatic energy with ligand binding (DE HBDesol), hydrophobic free energy generation (DE HPhobSolEl), hydrogen bond interactions (DE HBond Tor), hydrogen bridge donor-acceptor desolvation energy as described elsewhere [6].

### Pharmacokinetics Analysis:

ADMET (absorption, distribution, metabolism, excretion, toxicity) [1] properties were calculated using SwissADME (Figure 3 - see PDF) as described elsewhere [16].

## Results and Discussion:

The modified gedunin compound shown in Figure 1 (see PDF) is the ligand. The modified gedunin using ICM-Pro was selected after lowering the steric score and the ICM score is shown in Figure 2(see PDF). The modified gedunin showed an increased number of hydrogen and non-covalent bonds with low steric hindrance allowing easy binding of inhibitor inside the pocket of the enzyme. The results of the molecular docking [2] for the compound are given in Table 1 (see PDF). The lowest score shows effective binding of enzyme and inhibitor with the Hp score calculated as the difference between the conformation of the free ligand and the hydrophobic interaction energy found as -3.63, -4.743, -4.864 for acetyl cholinesterase, 5'nucleotidase, and metalloproteinase, respectively. It implies that 5'nucleotidase and metalloproteinase have the least hydrophobic interactions with the compound.

Table 2 (see PDF) shows the evaluation function for ligand enzyme complexes based on intermolecular interactions with hydrogen bond energy, van der Waals interaction energy (sum of gc and gh Vander Waals), hydrophobic energy when the surface is exposed to water, number of rotatable torsions, internal conformational energy of the ligand. Desolvation of exposed hydrogen-bond donors and acceptors is the change in the electrostatic solvation energy upon binding, mean force value of the ligand-receptor interaction strength [14]. Acetyl cholinesterase has the least negative (preferred) ICM score -14.98 followed by -7.035, -9.993 and PMF score: -51.33 followed by -29.36, -85.76 for 5' nucleotidase or metalloproteinase, all with ICM score <-15.0 and PMF score <-37.5. 5' nucleotidase and metalloproteinase - inhibitor complex is less stable than acetyl cholinesterase–inhibitor complex. The selection of the enzyme docking sites is based on the DLID likelihood for each pocket in enzymes. The DLID probability is highest in the active site of the enzyme. Table 3 (see PDF) shows the DLID, pocket buridness of enzymes. Pocket with a high DLID score is preferred as it has high drug likeliness in enzymes, which can be easily seen in molecular dynamics. Molecular dynamics simulation using GROMACS for 50 ns (nano-second) show that the average angle between Gedunin and 5'NT enzyme is between 70-760 with density between 980-984 kg/m3 increased due to complex formation.

The radius of gyration decreased from 1.2 to 1 after complex formation, implying tight bonding of complex. The number of hydrogen bonds between peptide-water is a maximum of 100-160, while between peptide-peptide their number is five. The binding energies decreased considerably to ∼ 0 Ki/mol, which shows the stability of the structure. Initially, RMSD was 8 nm, whereas, after the complex formation, RMSD is stable. The pressure is approximately 400 Bar and RMS is between 0.2-0.7. The overall simulation analysis shows that the molecule binds to the active center of 5'NT and the complex becomes stable. The Ramachandran plot analysis of all complex residues using Prove server with a mean z score of 0.931 is shown (Figure 8 - see PDF). Lipinski's rule of five determines the biological activity of the drug [10]. The total absorbed mass/dose of the drug is shown for the compound in Table 4 (see PDF). Table 6 (see PDF) also shows that ligand is a non-CNS drug calculated using SwissADME and medicinal chemistry analysis. Figure 3 (see PDF) shows the drug probability for the ligand.

Figure 4 (see PDF) shows a boiled egg diagram, with the yellow (egg yolk) region for the likelihood of brain penetration and the white region for passive absorption through the gastrointestinal tract. Table 5 (see PDF) shows that the ligand has broad substrate specificity and primarily through the phase 1 enzyme CYP Isoform 3A4 with a probability of 0.9691. Table 5 (see PDF) shows a probability of 0.66 according for isoform 3A4. The metabolism landscape shown in Figure 5 (see PDF) shows the selectivity of the ligand to isoform 3A4. Figure 5 (see PDF) shows that C25 and C27 are more labile than the rest of the ligand sites. The binding features for 2C9_Pki with hERG pic50 are shown in Table 6(see PDF). Table 7(see PDF) shows the Derek nexus probability [16] for the ligand with toxicity data [2,15]. The oral toxicity prediction is shown in (Figure 6 - see PDF) for Protox II, and the LD50 with 274 mg/kg. (Figure 7 - see PDF) shows the bioactivity of the R groups in the ligand using the luminous molecular property of Stardrop Software. Thus, these data help to describe the molecular docking analysis of gedunin (C26H31N2O6F) with enzymes (common in snake species) such as 5-nucleotidase, acetyl cholinesterase, L-aao, metalloproteinase, serine, thrombin and phospholipase A2 towards in combating snakebites.

## Conclusion:

We show the molecular docking analysis of gedunin (C26H31N2O6F) with enzymes (common in snake species) such as 5-nucleotidase, acetyl cholinesterase, L-aao, metalloproteinase, serine, thrombin and phospholipase A2. The modified gedunin in the enzyme pocket improves the pharmacological properties for further consideration in combating snakebites.

## Abbreviations:

PMF - peptide mass fingerprinting

LD50 - lethal dose 50

ADME - absorption, distribution, metabolism, and excretion

DLID - drug-like density

5'NT - 5'nucleotidase

RMSD - root mean square deviation

RMS - root mean square

CNS - center nervous system

TPSA - total polar surface area

CYP - cytochrome

## Author contributions:

Priya Dagar and Abha Mishra conceived the idea. Priya Dagar performed the computations. Abha Mishra verified the analytical methods and helped with the development of the manuscript.
